# The vital role of exercise and nutrition in COVID-19 rehabilitation: synergizing strength

**DOI:** 10.3389/fspor.2023.1305175

**Published:** 2023-12-08

**Authors:** Brent M. Peterson, Isabelle Unger, Sunny Sun, Ji-Yeun Park, Jinsil Kim, Richard S. Gunasekera, Jason Wilson, Thushara Galbadage

**Affiliations:** ^1^ Department of Kinesiology and Public Health, Biola University, La Mirada, CA, United States; ^2^ Department of Biological Sciences, Biola University, La Mirada, CA, United States; ^3^ Department of Chemistry, Physics, and Engineering, Biola University, La Mirada, CA, United States; ^4^ Department of Mathematics and Computer Science, Biola University, La Mirada, CA, United States

**Keywords:** COVID-19, exercise, nutrition, rehabilitation, intervention, Long-COVID, muscle strength, mental health

## Abstract

Since the outset of the COVID-19 pandemic, the global healthcare community has faced the challenge of understanding and addressing the ongoing and multi-faceted SARS-CoV-2 infection outcomes. As millions of individuals worldwide continue to navigate the complexities of post-hospitalization recovery, reinfection rates, and the increasing prevalence of Long-COVID symptoms, comprehensive COVID-19 rehabilitation strategies are greatly needed. Previous studies have highlighted the potential synergy between exercise and nutrition, suggesting that their integration into patient rehabilitation programs may yield improved clinical outcomes for survivors of COVID-19. Our group aimed to consolidate existing knowledge following the implementation of patient, intervention, comparison, and outcome (PICO) search strategies on the distinct and combined impacts of exercise and nutrition interventions in facilitating the recovery of COVID-19 patients following hospitalization, with a specific focus on their implications for both public health and clinical practice. The incorporation of targeted nutritional strategies alongside exercise-based programs may expedite patient recovery, ultimately promoting independence in performing activities of daily living (ADLs). Nonetheless, an imperative for expanded scientific inquiry remains, particularly in the realm of combined interventions. This mini-review underscores the compelling prospects offered by an amalgamated approach, advocating for the seamless integration of exercise and nutrition as integral components of post-hospitalization COVID-19 rehabilitation. The pursuit of a comprehensive understanding of the synergistic effects and effectiveness of exercise and nutrition stands as a crucial objective in advancing patient care and refining recovery strategies in the wake of this enduring global health crisis.

## 1. Introduction

Since initial cases were identified in November 2019, the global healthcare community has contended with the relentless challenge posed by the coronavirus disease (COVID-19), caused by the severe acute respiratory syndrome coronavirus 2 (SARS-CoV-2) (1). By some reports, COVID-19 has become endemic (2), however, recent statistics paint a sobering picture. Approximately 4.5 million new cases and 32,000 deaths occurred between February 6th and March 5th, 2023 (3). As of March 5th, 2023, a staggering 759 million confirmed cases and 6.8 million deaths have been reported worldwide, emphasizing the enduring need for public health and medical interventions (3). As COVID-19 transitions into an endemic disease, the inevitable consequence of a growing survivor population has become apparent.

An increasing number of adults, regardless of vaccination status, have been contending with an array of lingering symptoms and post-acute conditions collectively known as post-COVID conditions, long-haul COVID, or more commonly noted as long-term COVID (4); more so in older adults, ages 65 and older (5). While symptom severity varies, most patients encounter the triad of coughing, fever, and fatigue, often accompanied by anosmia, headaches, myalgia, congestion, rhinorrhea, dyspnea, vomiting, or diarrhea, which may worsen a week or more after onset (6). Persistent symptoms lasting longer than four weeks following diagnosis, and some suffering effects for greater than two years have been reported (7). While some COVID-based physical rehabilitation studies exist to address these issues, to our knowledge, no studies have examined the implementation of exercise and nutritional interventions in a COVID rehabilitation model. Thus, the purposes of this mini-review were: (1) to evaluate and synthesize existing knowledge regarding the pivotal role of exercise or nutritional interventions for COVID patients, and (2) to propose COVID rehabilitation modeling which includes a combination of exercise and nutritional interventions aimed at optimal recovery, physical well-being, and improved quality of life (QOL). Search strategies employed by our group followed the patient, intervention, comparison, and outcome (PICO) model (Supplementary Table S1) (8).

## 2. Impact of COVID-19 on patients’ health and significance of rehabilitation

Hospitalized or recovering patients may experience substantial mental health challenges directly from the disease, which often overlap with physical challenges, and then contribute to the overall complexity of psychological well-being (9, 10). Hospitalization, isolation, and uncertainty in severe illness can precipitate mental health challenges or post-traumatic stress disorder (PTSD)-like symptoms (11–13), cognitive disorders, memory lapses, and difficulties in concentrating (14). Immunological dysregulation (15), ongoing inflammatory conditions (16, 17), and organ system dysfunction have been linked to symptom persistence (18). Persistent anxiety and depression may result from the uncertainties surrounding the illness, isolation, and exposure to illness and mortality (19). Cognitive challenges, described as brain fog, may further complicate recovery (20). Rehabilitation for patients who continue to experience lingering symptoms is paramount as it may vitally impact the recovery process by improving outcomes, reducing complications, and enhancing overall QOL (21). Muscle weakness, decreased mobility, and reduced functional capacity have been highlighted as a consequence of prolonged bed rest and the catabolic effects of the virus (22). Persistent fatigue further exacerbates the challenges faced by patients, hampering their ability to engage in daily activities and threatening overall QOL (23, 24). Additionally, the virus's potential to influence the development of novel health conditions, such as metabolic and cardiovascular issues, adds another layer of complexity to the rehabilitation process (25). The Stanford-Hall consensus statement for post-COVID rehabilitation, while initially targeting active individuals, has provided a valuable rehabilitative framework for addressing the major health challenges faced by COVID-19 patients (25). However, nutritional interventions were notably absent from this consensus, leaving an important aspect of recovery unaddressed.

## 3. Exercise in COVID-19 rehabilitation

Human movement, whether by physical activity or exercise, has been long established in the literature for the wide array of health benefits and for the reduction of risks of developing chronic health disorders or disease states. Ruegsegger and Booth (2018), in a review of the literature, reported that lifelong exercise is associated with the delayed onset of approximately 40 different diseases in addition to the extenuation of lifespan (26). Numerous studies have demonstrated improvements in cardiovascular and cardiorespiratory function (27–29), improvements in mental health and QOL (30–34), and improvements in blood parameters, mitochondrial function, and metabolic conditions (35–38).

Contemporary rehabilitation strategies have been developed and implemented for large-scale health conditions such as cancer and cardiovascular disease according to phase-based standard-of-care models (39, 40). Both follow progressively similar trajectories often initiating in the in-patient setting to address medical or surgical issues with goals of referring patients toward prescriptive out-patient rehabilitation programs. Assessment and screening procedures guide patient allocation toward addressing specific needs of symptom reduction and improvements in functional capacity. Then, as patient progress and physiological improvement occurs, exercise interventions advance through additional phase increases resulting in an individual maintaining healthy active living and independence at the completion of the final phase (41). Comparitively, phase-based cancer rehabilitation modeling may be more appropriate because of the greater expectance of a wide array of known cancer treatment-related side effects such as chemotherapy-related cognitive impairment, balance and coordination disorders, fatigue, muscle and joint pain, depression, reductions in QOL, urinary or gastrointestinal disorders, thrombocytoppenia, integumentary system disorders, neuropathic conditions, cardiorespiratory conditions, fertility issues, and edema (42, 43).

## 4. Nutrition in COVID-19 rehabilitation

Infection susceptibility and advanced disease severity have been linked to a number of risk factors including male gender, advanced age, obesity, type-II diabetes diagnoses, poor diet, under-represented population groups, and pro-inflammatory conditions (44, 45). Butler and Barrientos (2020) suggested that highly processed diets composed of high fat and carbohydrate content, low fiber foods may play a significant role in not only association with risk factors but also with negative impacts on innate and adaptive immunological function (46). Excessive dietary sodium and alcohol in addition to deficiencies in vitamin D may also negatively predispose an individual to be at greater risk for infection and greater severity of the disease (47). Yet, Downer, Berkowitz, Harlan, Olstad, and Mozaffarian, et al. (2020) suggest that targeted nutritional interventions have a significant role in the treatment, prevention, management, and reversal of some diseases (48). As it relates directly to COVID-19, Tsagari, Risvas, Papathanasiou, and Dionyssiotis (2022) specifically noted that patients are more prone to sarcopenia and malnutrition due to multiple nutritional and metabolic factors that may result in decreases in immune function, decreases in respiratory and swallowing function, and reductions in resilience to metabolic stress. Yet, a consensus would indicate that obtaining macro- and micronutrients in proper concentrations is conducive to overall health and well-being (49).

## 5. The synergy of exercise and nutrition

Adequate nutrition necessary for healthy human function has been demonstrated to support immunological function, organ health, and functioning, prolong lifespans, and decrease risks for diseases such as diabetes and some cancers (50). In addition to the benefits of exercise during patient rehabilitation, adequate nutrition complements positive outcomes and provides potential synergistic strength when they are utilized in combination (Table 1). Given the abundance of evidence supporting the positive impacts of exercise or nutrition alone, the combined exercise and nutrition theoretical construct synergizes both for potentially additive effects. Yet, research is often convoluted and challenging to conduct because of the many different factors presented herein. Thus, our group strongly advocates for increased research in this area with the hopes that ultimately, these investigative efforts may lead to wide-scale development and implementation of standardized exercise and nutrition programs that are targeted toward the individual needs of patients recovering from COVID-19.

**Table 1:** Benefits of exercise, nutrition, and their synergistic effects during patient rehabilitation and recovery.

Rehabilitation Benefit	Importance in Patient Rehabilitation	References
Exercise		
Respiratory function	Helps strengthen the respiratory muscles and improve lung capacity	(40, 51)
Muscle strength and mobility	Prevents muscle atrophy and enhances muscle strength and mobility	(22, 51)
Cardiovascular health	Contributes to better cardiovascular health and reduces the risk of heart-related complications	(36, 40, 52)
Mental health	Releases endorphins, which can alleviate symptoms of anxiety and depression	(31, 34, 53)
Weight management	Reduces the risk of complications in individuals with obesity-related comorbidities	(44, 54)
Balance and coordination	Helps regain stability and reduce the risk of falls in high-risk patients.	(22, 51)
Nutrition		
Immune support	Provides essential nutrients that bolster the immune system, aiding in the body's ability to fight infections	(16, 46, 49, 50, 55)
Tissue repair and healing	Supports tissue repair and healing processes, aiding in the recovery from the physical impact of the disease	(56, 57)
Energy and strength	Provides energy for daily activities, and rehabilitation exercises, and prevents muscle wasting and fatigue	(50, 56, 57)
Bone health	Adequate calcium and vitamin D intake contribute to bone strength and can aid in the healing process	(49, 50, 57)
Inflammation	Reduces systemic inflammation and contributes to lower disease-related complications	(16, 49, 56, 57)
Mental health	Supports cognitive function and emotional health during the post-disease recovery period	(49, 58)
Combined Exercise and Nutrition		
Muscle preservation and growth	Help preserve existing muscle mass and promote the development of lean muscle tissue. Resistance training stimulates muscle protein synthesis. Protein intake provides the necessary building blocks for muscle repair and growth.	(40, 51, 56)
Exercise performance	Lead to more effective workouts and greater gains in strength and mobility. Carbohydrate intake provides the energy required for physical activity. Consuming the right nutrients before and after exercise can help perform better during rehabilitation sessions.	(51, 52, 56)
Enhanced recovery	Nutrients including vitamins, minerals, and antioxidants aid in tissue repair and reduce inflammation. When combined with appropriate exercise, these nutrients support an accelerated healing and recovery process.	(40, 51, 52, 56)
Immune function	Regular exercise can temporarily suppress the immune system. A well-balanced diet supports immune responses, helping the body fight off infections, including those that can interfere with rehabilitation.	(21, 51, 55, 56)
Weight management	Exercise increases energy expenditure, while proper nutrition helps control calorie intake. They create an energy balance that can support weight loss, weight maintenance, or healthy weight gain.	(21, 44, 54, 56)
Mental health	Promote overall mental health and well-being during rehabilitation and recovery. Exercise reduces stress and improves mood. Foods rich in omega-3 fatty acids and antioxidants can have a positive impact on mood and cognitive function.	(13, 34, 53, 58)
Risk of comorbidities	Address underlying patient health conditions and comorbidities these conditions, reducing the risk of complications and promoting better overall health.	(21, 40, 52, 56, 59)
Nutrient utilization	Improve nutrient utilization and contribute to better overall health and recovery. Exercise increases the body's demand for nutrients. Nutrients are more efficiently utilized to support recovery during rehabilitation.	(49, 51, 56)
Patient compliance	When patients see positive results from their rehabilitation efforts, they are more likely to stay motivated and adhere to their exercise and nutrition plans. The synergy between exercise and nutrition can lead to faster improvements, boosting patient confidence and compliance.	(21, 51, 56)
Quality of life	Exercise and nutrition contribute to an improved quality of life by enhancing physical and mental health, reducing symptoms, and increasing patients’ functional capacity.	(14, 20, 30, 33, 51, 56)

Patient education on diet and exercise may also substantially impact COVID-19 rehabilitation by accelerating the return to functional capacity by empowering individuals to take an active role in their recovery. A combined holistic approach that integrates targeted exercise regimens with nutritional guidance may provide additional effectiveness. Nutritional interventions support the healing process, enhance energy levels, and aid in muscle recovery, complementing the benefits of physical rehabilitation (60). Emphasizing the importance of exercise and nutrition in rehabilitation, may reduce the long-term burdens of COVID-19 and help patients regain their independence and overall well-being.

## 6. Discussion

**Figure 1:**
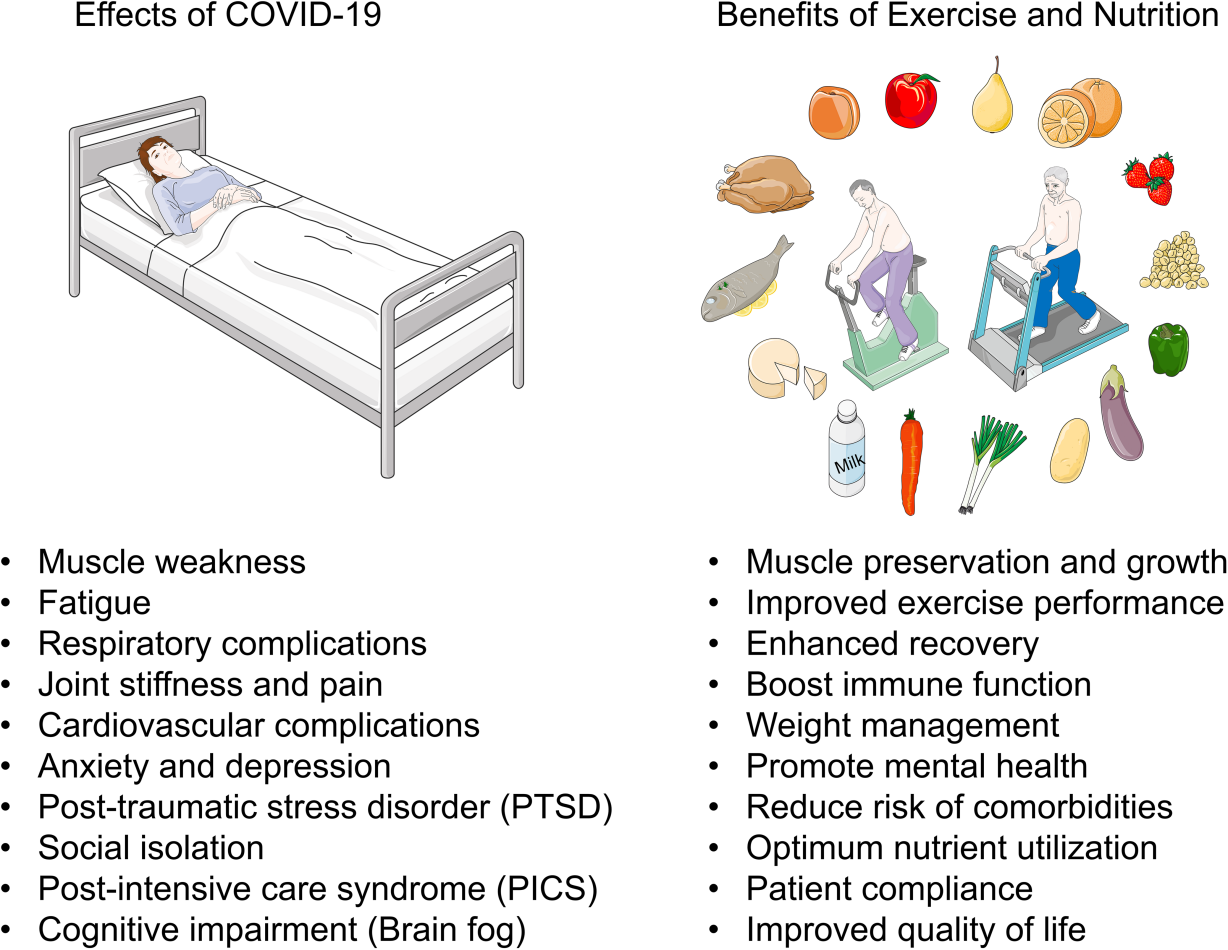
Benefits of exercise and nutritional interventions for patients hospitalized with COVID-19. Patients hospitalized with COVID-19 may experience symptoms including, muscle weakness, fatigue, respiratory complications, joint stiffness and pain, cardiovascular complications, anxiety and depression, post-traumatic stress disorder (PTSD), social isolation, post-intensive care syndrome (PICS), and cognitive impairment (brain fog). Incorporating exercise and nutritional interventions during patient rehabilitation could elicit synergistic benefits including, muscle preservation and growth, improved exercise performance, enhanced recovery, boost immune function, weight management, promote mental health, reduce risk of comorbidities, optimum nutrient utilization, patient compliance, and improved quality of life. This figure utilizes images available at Servier Medical Art, licensed under a Creative Commons Attribution 3.0 Unported License.

The implementation of combined exercise and nutritional interventions in a hospitalized setting for patients with COVID-19 presents with several challenges (Figure 1). COVID-19 not only affects the respiratory system but also leads to muscle weakness, deconditioning, and nutritional deficiencies due to prolonged illness and reduced food intake. Integrating exercise and nutrition therapies is crucial for addressing both the respiratory and musculoskeletal aspects of recovery. Such an approach not only enhances physical function but also supports mental and emotional well-being during the long recovery process. One significant obstacle is ensuring patient compliance, particularly in cases of severe illness or limited mobility. Patients may experience discomfort or fatigue, making it difficult to engage in exercise routines. Additionally, resource limitations in healthcare facilities, such as a shortage of qualified personnel, equipment, or dedicated spaces for exercise, can hinder program implementation. Overcoming these challenges requires a multifaceted approach with a multidisciplinary team who are trained and equipped to address this disease from the acute phase throughout active recovery and return to apparently healthy functional status. Clinical exercise physiologists, physiotherapists, dietitians, occupational therapists, and statisticians working alongside physicians, nurses, and specialists play essential roles in the assessment, development, monitoring, and in-depth analytical evaluations of programmatic implementation specifically targeting patient-specific needs and conditions throughout the continuum of care. Personalized expert guidance may increase the likelihood of intervention adherence.

As we continue to navigate the complex landscape of rehabilitation for hospitalized patients, especially those with COVID-19, there are promising avenues for future health education, research, and innovative methodologies to address disease risk, infection, hospitalization, recovery, and rehabilitation. Investigating the optimal timing and intensity of exercise interventions, along with personalized nutrition plans, can further improve patient outcomes. The integration of technology, such as telehealth platforms and wearable telemetric devices, may facilitate remote monitoring and enhance patient engagement. Moreover, research should explore the long-term effects of combined exercise and nutrition therapy on reducing the risk of readmission, improving overall quality of life, and addressing potential comorbidities associated with sarcopenia and malnutrition.

The successful implementation of exercise and nutrition programs in a hospital setting, particularly for patients recovering from severe illnesses like COVID-19, necessitates a comprehensive strategy. Overcoming patient compliance issues, and resource constraints, and ensuring a multidisciplinary approach are critical steps. Future research endeavors and innovative approaches should aim to refine these interventions, ultimately leading to better patient outcomes and improved healthcare delivery.
