# Supplementary material for: The vital role of exercise and nutrition in COVID-19 rehabilitation: synergizing strength
Source: Front Sports Act Living. 2023 Dec 8;5:1305175. doi: 10.3389/fspor.2023.1305175 (PMC10748488; doi:10.3389/fspor.2023.1305175)
Supplement: Supplementary file 1 [file Table1.docx]

**The Vital Role of Exercise and Nutrition in COVID-19 Rehabilitation: Synergizing Strength**

**Supplemental Information**

Brent M. Peterson^1*^, Isabelle Unger^1^, Sunny Sun^1^, Ji-Yeun Park^1^, Jinsil Kim^2^, Richard Gunasekera^3^, Jason Wilson^4^, & Thushara Galbadage^1*^

^1^ Department of Kinesiology and Public Health, Biola University, ^2^ Department of Biological Sciences, ^3^ Department of Chemistry, Physics, and Engineering, ^4^ Department of Mathematics and Computer Science, La Mirada, CA, United States

**Supplemental Table 1.** Population, Intervention, Comparator, Outcome (PICO) literature search strategy categories.

| Category | Description |
| --- | --- |
| Study Title | The Vital Role of Exercise and Nutrition in COVID-19 Rehabilitation: Synergizing Strength. |
| Population | Adult men and women of all demographics who were hospitalized for COVID-19. |
| Intervention | Rehabilitation protocols that include exercise, nutrition, or exercise and nutrition employed in the inpatient, outpatient, or both settings for COVID-19 patients. |
| Comparator | Standard of care, or before and after rehabilitation. |
| Outcome | Changes in physiological function, mental health, cognitive function, quality of life, well-being, and alleviation of symptoms related to COVID-19. |
